# Immune profile of the tumor microenvironment and the identification of a four-gene signature for lung adenocarcinoma

**DOI:** 10.18632/aging.202269

**Published:** 2020-12-09

**Authors:** Tao Fan, Mingchuang Zhu, Liyu Wang, Yu Liu, He Tian, Yujia Zheng, Fengwei Tan, Nan Sun, Chunxiang Li, Jie He

**Affiliations:** 1 Department of Oncology, Renmin Hospital of Wuhan University, Wuhan 430060, China; 2 Department of Thoracic Surgery, National Cancer Center/National Clinical Research Center for Cancer/Cancer Hospital, Chinese Academy of Medical Sciences and Peking Union Medical College, Beijing 100021, China

**Keywords:** lung adenocarcinoma, tumor microenvironment, tumor immunity, tumor stroma, immune cell infiltration

## Abstract

The composition and relative abundances of immune cells in the tumor microenvironment are key factors affecting the progression of lung adenocarcinomas (LUADs) and the efficacy of immunotherapy. Using the cancer gene expression dataset from The Cancer Genome Atlas (TCGA) program, we scored stromal and immune cells for tumor purity prediction by CIBERSORT and ESTMATE. Differential expression analysis was employed to identify 374 genes between the high-score group and the low-score group, which were utilized to conduct Gene Ontology (GO) and Kyoto Encyclopedia of Genes and Genomes (KEGG) enrichment analysis. Protein-protein interaction (PPI) and Cox regression analysis were performed on the differentially expressed genes (DEGs) to identify four key tumor microenvironment (TME) -related genes (CCR2, CCR4, P2RY12, and P2RY13). The expression levels of the four DEGs differed significantly among LUAD patients of different ages, genders, and TNM stages. We found that the infiltration of resting memory CD4+ T cells, memory B cells, and M0 macrophages into the TME was co-regulated by these four DEGs. These four genes were closely related to the prognosis of LUAD and affected the infiltration of immune cells into the TME, which had predictive prognostic value in LUAD.

## INTRODUCTION

Lung cancer is the most common cancer worldwide, and it is one of the leading causes of cancer death [1, 2]. Lung adenocarcinoma is a major type of lung cancer. The human immune system is the natural enemy of tumor cells. In recent years, tumor immunotherapy has gradually developed into one of the primary methods for treating tumors [3, 4]. Many major breakthroughs have been achieved in utilizing immunotherapy to treat lung adenocarcinoma [5–7]. The immune system is the first line of defense against diseases. Single-cell analysis has been applied to detect changed T cell and NK cell compartments in lung adenocarcinoma [8]. Tumor-driven immune imbalance is likely to be a key factor affecting tumor development and progression. The tumorigenic process is highly complex, and it has been confirmed that immune cells are involved in many aspects of tumor formation [9–11].

As a novel cancer treatment strategy, immunotherapy remains limited to a relatively small proportion of cancer patients, although it has been employed to achieve satisfactory results in tumor treatment [12]. The efficacy of immunotherapy is closely related to the tumor microenvironment (TME). Tumor cell epigenetic differentiation and infiltration metastasis are associated with tumor-induced immune suppression. The TME is an intricate system that includes various types of cells, cytokines and other extracellular components. The type and number of infiltrating immune cells are important in determining tumor occurrence and progression. The composition and proportion of tumor-infiltrating immune cells (TIICs) and stroma can also be utilized for the prognosis and prediction of various types of cancer [13–15]. For example, intraepithelial CD8-positive and PD1-positive tumor-infiltrating lymphocytes, as defined by CD103, have prognostic significance in endometrial adenocarcinoma [16]. Increased CD8 infiltration is associated with impaired progression-free survival (PFS) and overall survival (OS). Patients with high CD8+ T cell density often exhibit high expression of PD-L1, which indicates that adaptive immune resistance may occur in the tumor microenvironment [17]. Inhibition of the IL-9 or IL-17 cytokines can reduce epithelial-mesenchymal transition (EMT) and slow the progression and metastasis of lung cancer [18]. While some TIICs kill tumor cells, some tumor-associated macrophages (TAMs) help tumor cells escape the body's immunity to promote tumor development. STAT-6 promotes the pro-tumoral M2-like phenotype of TAMs in advanced-stage EMT by upregulating the expression of immune suppression genes and tumor stromal remodeling [19]. As another cell type in the TME, stromal cells have a bidirectional and complex relationship with tumor cells. It has noted that carcinoma-associated fibroblast (CAF)-derived exosomes induce lung pre-metastatic niche formation and increase lung metastasis [20]. In addition, CAFs may block the delivery of drugs and induce drug resistance, which are significant factors that may contribute to the poor prognosis of cancer patients [21, 22]. Mining related genes and subsequently studying their impact on TME immune cell infiltration, as well as on tumor prognosis, may provide new targets for tumor treatment.

In this study, we took advantage of the TCGA dataset, including 551 transcriptome profiles and 486 clinical datasets. Subsequently, ESTIMATE software was employed to score the immune and stromal cells in each sample. After GO and KEGG enrichment analysis, PPI analysis, Cox regression analysis, and correlation analysis of immune infiltrating cells and gene expression were performed, four genes (CCR2, CCR4, P2RY12, and P2RY13) were finally identified;, these genes were determined to co-regulated the infiltration of M0 macrophages, resting memory CD4+ T cells, and memory B cells in the TME of LUAD. We found that low expression levels of these four genes correlated with poor clinical prognosis and infiltration of tumor immune cells in LUAD. These defined immune-related genes with potential prognostic value provide a new method for predicting the progression of LUAD and provide ideas for the development of novel immunotherapies for LUAD.

## RESULTS

### Tumor microenvironment score is associated with prognosis, age, gender, and TNM stage of LUAD patients

**Figure 1:**
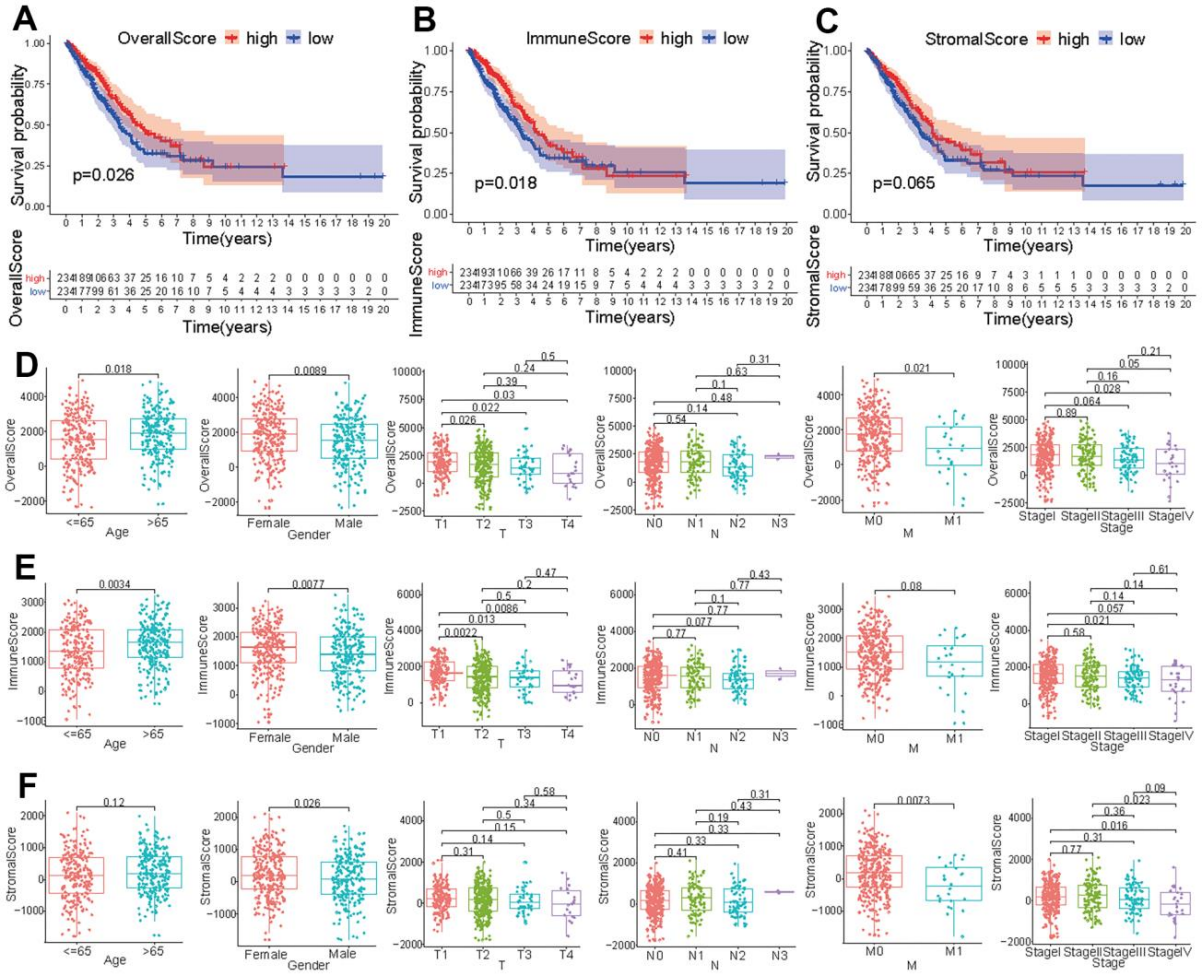
**Correlation of tumor score with different clinical features.** Survival analysis of patients with LUAD based on overall score (**A**), immune score (**B**), and stromal score (**C**). Effect of age, gender or tumor TNM stage on overall score (**D**), immune score (**E**), and stromal score (**F**).

In order to identify the immune characteristics of the tumor microenvironment of LUAD and screen prognostic-related genes, an analysis process was established to show the screening process (Supplementary Figure 1). After analyzing the FPKM data from TCGA, we divided the immune and stromal cells in each sample into high-score and low-score groups. LUAD patients with a high overall score or immune score presented a longer survival time than did those with a low score (*p*<0.05) (Figure 1A, 1B). However, the survival time of patients with high stromal scores was not significantly different from that of patients with low stromal scores (*p* > 0.05) (Figure 1C). The overall score in tumor samples differed between ages < 65 and > 65 and varied by gender (Figure 1D). The overall score in T1 patients was different from that in obtained in T2, T3, and T4 patients, and the overall score in M0 patients was different from that in M1 patients (Figure 1D). Except for the significant difference in overall score observed between stage I and stage IV, there were no significant differences among other clinical stages (Figure 1D). The immune score in tumor samples was significantly different between ages < 65 and > 65 and varied by gender (Figure 1E). Immune scores in T1 patients were different from those in T2, T3, and T4 patients (Figure 1E). Except for the significant difference in immune score between stage I and stage III, there were no significant differences among other clinical stages (Figure 1E). The stromal score in tumor samples differed significantly between males and females and varied across different M stages (Figure 1F). Although the stromal score of stage IV patients was different from that of stage I and stage II patients, there were no significant differences among the other clinical stages (Figure 1F).

### Obtaining DEGs and performing enrichment analysis

**Figure 2:**
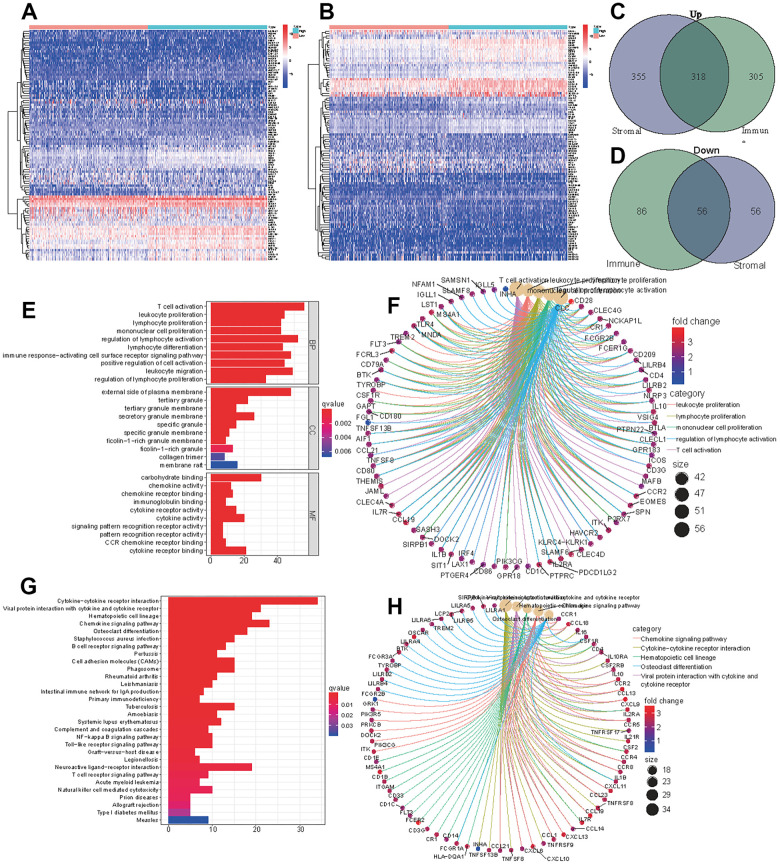
**DEGs of high immune score (stromal score) and low score groups and functional enrichment analysis.** Heatmap of significantly differentially expressed genes based on immune (**A**) and stromal (**B**) scores for LUAD. Venn diagram analysis of high (**C**) and low (**D**) expressed genes based on immune and stromal scores. (**E**) GO analysis of aberrantly expressed genes at the intersection of two groups. (**F**) CircleMap showing the functional interactions between pathways and genes as extracted from GO. (**G**) KEGG analysis of aberrantly expressed genes at the intersection of two groups. (**H**) CircleMap showing the functional interactions between pathways and genes as extracted from KEGG.

The samples were divided into a high immune (or stromal) score group and a low immune (or stromal) score group. A total of 623 upregulated genes and 142 downregulated genes were screened from the high immune score group, and 673 upregulated genes and 112 downregulated genes were screened from high stromal score group (Figure 2A, 2B). We obtained 318 upregulated genes and 56 downregulated genes by taking the intersection of the DEGs in the two groups (Figure 2C, 2D). All 374 DEGs were further employed to conduct GO (Figure 2E, 2F) and KEGG (Figure 2G, 2H) enrichment analyses to elucidate their functions in tumorigenesis and progression. T cell activation, leukocyte proliferation and lymphocyte proliferation were the most enriched pathways, and most genes were related to immune- or stromal- cell activation and proliferation. These TME-related genes and cells are the primary causes of tumor growth and deterioration.

### Screening of the most important DEGs

**Figure 3:**
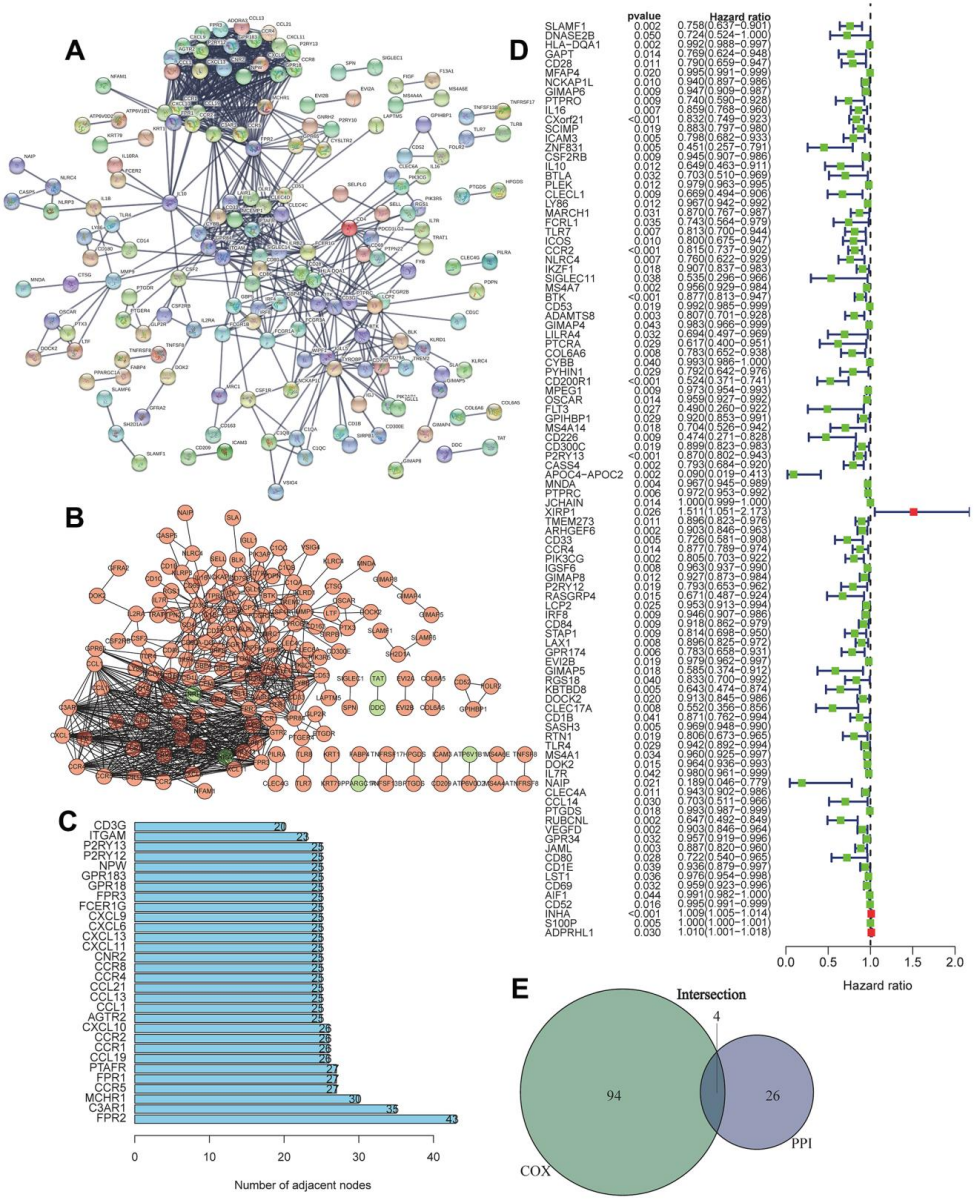
**Screening of differentially expressed genes based on protein-protein interaction (PPI) and Univariate Cox regression analysis.** (**A**) PPI network of the aberrantly expressed genes based on STRING (interaction confidence value > 0.95). (**B**) Visualized PPI analysis of differentially expressed genes using Cytoscape. (**C**) Top 30 genes with maximum adjacent nodes. (**D**) Univariate Cox regression analysis for the aberrantly expressed genes. Genes with a *p* value less than 0.05 are shown in the forest plot. (**E**) Venn diagram of key genes in PPI and Cox regression analysis. Four TIIC-related genes (CCR2, CCR4, P2RY12, and P2RY13) were finally screened as prognostic factors of LUAD.

We first conducted PPI network analysis based on 374 DEGs using STRING (Figure 3A) and Cytoscape software (Figure 3B). A total of 371 nodes and 732 edges were identified from PPI network analysis of 374 immune-related DEGs (minimum required interaction score > 0.9). The top 30 proteins that had the maximum number of nodes in the PPI network are displayed in Figure 3C. We further conducted Univariate Cox regression analysis on 374 immune-related DEGs, and 98 prognostic genes were identified as risk factors for LUAD (Figure 3D). The four core target genes (CCR2, CCR4, P2RY12, and P2RY13) were screened out by taking the intersection of the top 30 genes and 98 candidate prognostic genes (Figure 3E).

### Expression levels and survival curve of the four target genes in LUAD

**Figure 4:**
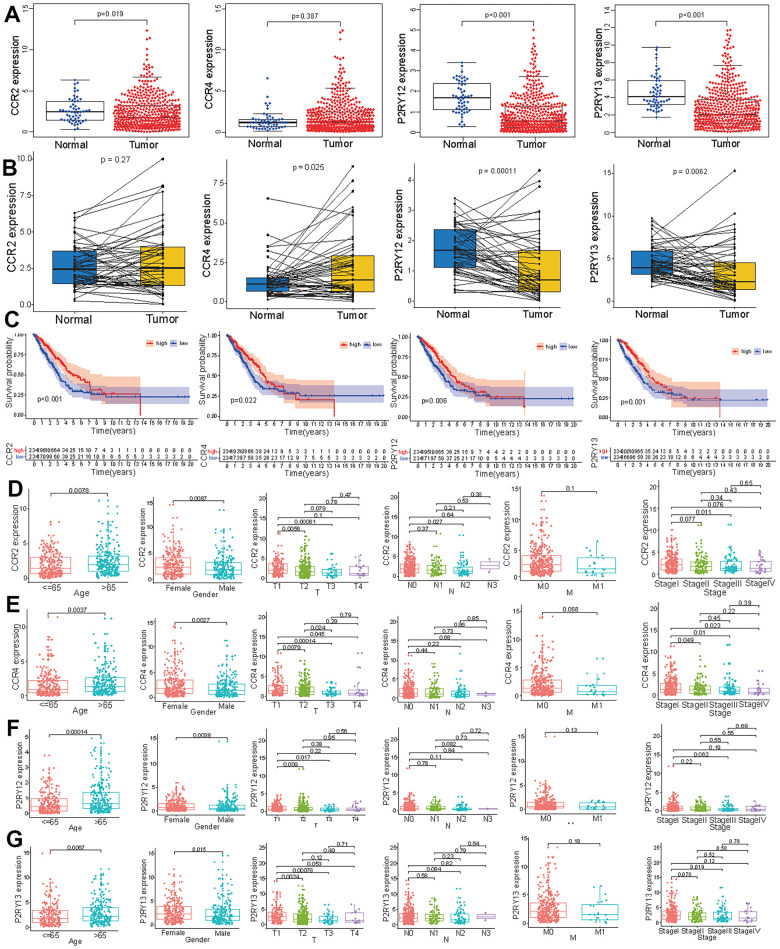
**Expression levels of the four genes (CCR2, CCR4, P2RY12, and P2RY13) and their prognostic value in LUAD patients.** (**A**) The expression levels of the four genes in LUAD and normal tissues. (**B**) The levels of these four genes in paired tumor and adjacent normal tissues. (**C**) Survival curves of the expression of these four genes in the high-expression (red line) and low-expression (blue line) groups. The expression levels of CCR2 (**D**), CCR4 (**E**), P2RY12 (**F**), and P2RY12 (**G**) in patients with LUAD of different ages, genders and tumor TNM stages.

To verify the effect of these four genes on LUAD, we measured the expression levels of the four genes in tumor tissues and normal tissues. The expression levels of CCR2, P2RY12, and P2RY13 in tumor tissues were significantly lower than those observed in normal tissues, and the expression level of CCR4 did not differ between tumor tissues and normal tissues (Figure 4A). We further compared the expression levels of these four genes in paired tumor and adjacent normal tissues, which showed that the expression levels of CCR4, P2RY12, and P2RY13 in tumor tissues were significantly lower than those in adjacent normal tissues, and the expression level of CCR2 did not differ between paired tumor and adjacent normal tissues (Figure 4B). To further study the impact of these four genes on survival time, LUAD patients were divided into a high-expression group and a low-expression group based on the expression levels of the four genes. The results indicated that LUAD patients with high expression levels of the four genes had a better prognosis and higher survival time (Figure 4C). We also investigated the distribution of the expression of these four genes across different ages, genders and TNM stages. The CCR2 expression level in female LUAD patients or patients aged > 65 was higher than that in male LUAD patients or patients aged ≤ 65(Figure 4D) (*p* < 0.05). The CCR2 expression level in T1 patients was higher than that in T2 and T3 patients (Figure 4D) (*p* < 0.05). The CCR2 expression level in N0 patients was higher than that observed in N2 patients (Figure 4D) (*p* < 0.05). Except for the finding that the CCR2 expression levels in LUAD patients with stage I disease were higher than those in LUAD patients with stage III disease, no difference appeared between patients with other clinical stages of disease (Figure 4D) (*p* < 0.05). The CCR4 expression level in female LUAD patients or patients aged > 65 was higher than that in male LUAD patients or patients aged ≤ 65(Figure 4E) (*p* < 0.05). The CCR4 expression level in T1 patients was different from that in T2, T3, and T4 patients, and the CCR4 expression level in T2 patients was different from that in T3 patients (Figure 4E) (*p* < 0.05). The CCR4 expression level in LUAD patients with stage I disease was higher than that in patients with stage II, stage III, and stage IV disease (Figure 4E) (*p* < 0.05). The P2RY12 expression level in female LUAD patients or patients aged > 65 was higher than that in male LUAD patients and patients aged < 65(Figure 4F) (*p* < 0.05). Except for the finding that P2RY12 expression levels in T1 patients were higher than those in T2 and T3 patients, no difference was observed between patients with other clinical TNM stages (Figure 4F) (*p* < 0.05). The P2RY13 expression level in female LUAD patients or patients aged > 65 was higher than that in male LUAD patients or patients aged < 65(Figure 4G) (*p* < 0.05). P2RY13 expression levels in T1 patients were different from those in T2 and T3 patients (Figure 4G) (*p* < 0.05). Except for the finding that P2RY13 expression levels in LUAD patients with stage I disease were higher than in those with stage III disease, no differences between other clinical stages were observed in patients (Figure 4G) (*p* < 0.05).

### GSEA for the four genes

**Figure 5:**
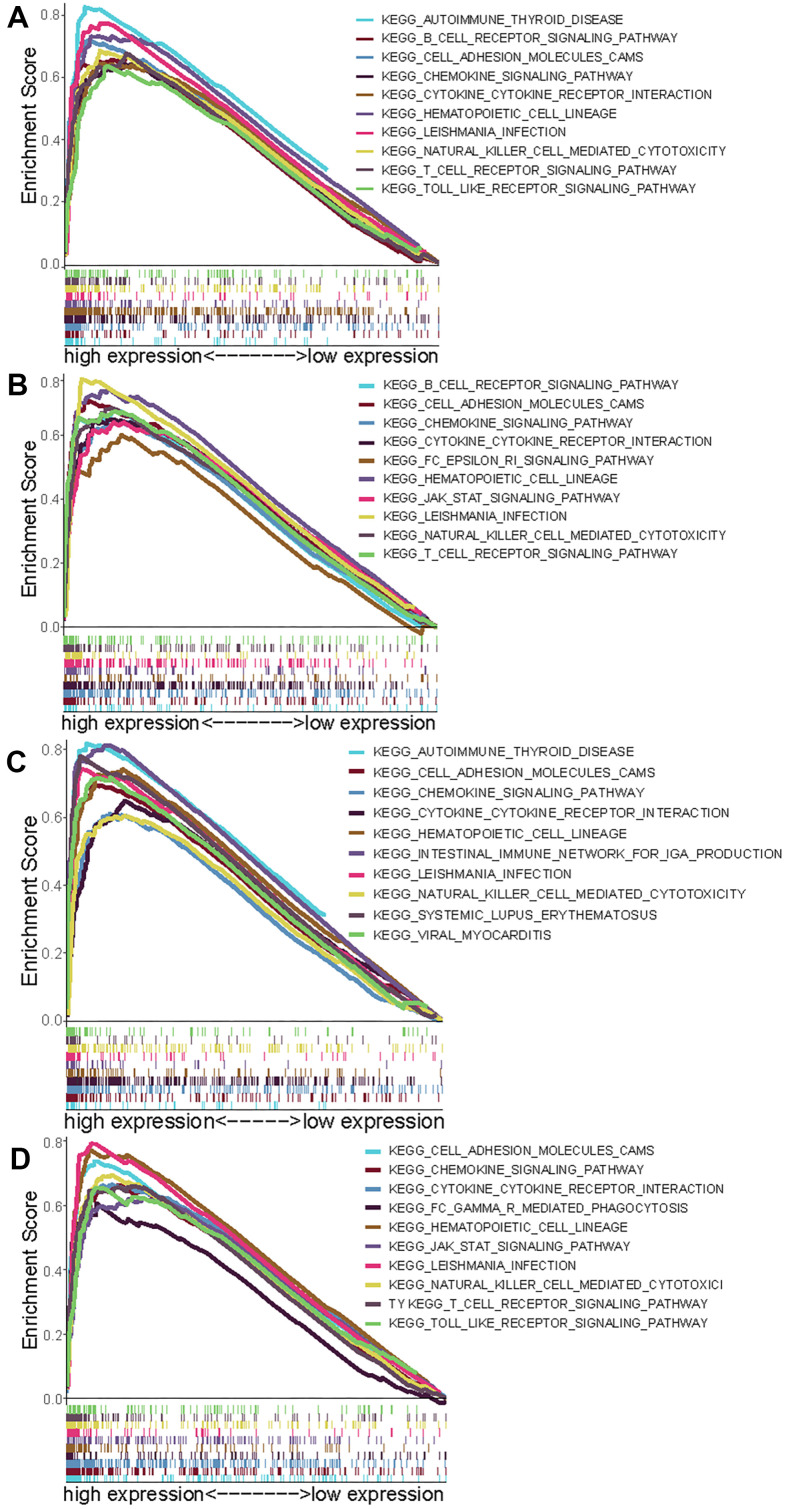
**Select GSEA plots of signatures for the four genes.** (**A**) Enriched gene sets in KEGG collection by high expression of CCR2 (**A**), CCR4 (**B**), P2RY12 (**C**), and P2RY13 (**D**). Each line with a unique color represents one particular gene set. The upregulated genes are located on the left of the x-axis, and the downregulated genes are on the right. Only the gene sets with FDR *q* < 0.05, NOM *p* < 0.05, and FWER *p* < 0.05 are displayed. The top 10 leading gene sets are presented in the plot.

To research signaling pathways related to these four genes (CCR2, CCR4, P2RY12, and P2RY13), gene set enrichment analysis (GSEA) was performed to select significantly enriched signaling pathways according to NES, nominal *p*-value, FDR *q*-value and FWER *p*-value. The 10 most important signaling pathways enriched in highly expressed phenotypes of CCR2 were autoimmune disease, B cell receptor signaling pathway, cell adhesion molecules, chemokine signaling pathway, cytokine- cytokine interaction, hematopoietic cell lineage, Leishmania infection, NK cell-mediated cytotoxicity, T cell signaling pathway, and Toll-like receptor signaling pathway (Figure 5A). The 10 most important signaling pathways enriched in highly expressed phenotypes of CCR4 were the B cell receptor signaling pathway, cell adhesion molecules, cytokine- cytokine interaction, Fc epsilon RI signaling pathway, hematopoietic cell lineage, JAK-STAT signaling pathway, Leishmania infection, NK cell-mediated cytotoxicity, and T cell receptor signaling pathway (Figure 5B). The 10 most important signaling pathways enriched in highly expressed phenotypes of P2RY12 were autoimmune disease, cell adhesion molecules, chemokine signaling pathway, cytokine- cytokine interaction, hematopoietic cell lineage, intestinal immune network for IgA production, Leishmania infection, NK cell-mediated cytotoxicity, systemic lupus erythematosus, and viral myocarditis (Figure 5C). The 10 most important signaling pathways enriched in highly expressed phenotypes of P2RY13 were cell adhesion molecules, chemokine signaling pathway, cytokine- cytokine interaction, FcγR -mediated phagocytosis, hematopoietic cell lineage, JAK-STAT signaling pathway, Leishmania infection, NK cell-mediated cytotoxicity, T cell receptor signaling, and toll like receptor signaling pathway (Figure 5D).

### Distribution characteristics of immune cells in the TME of LUAD

**Figure 6:**
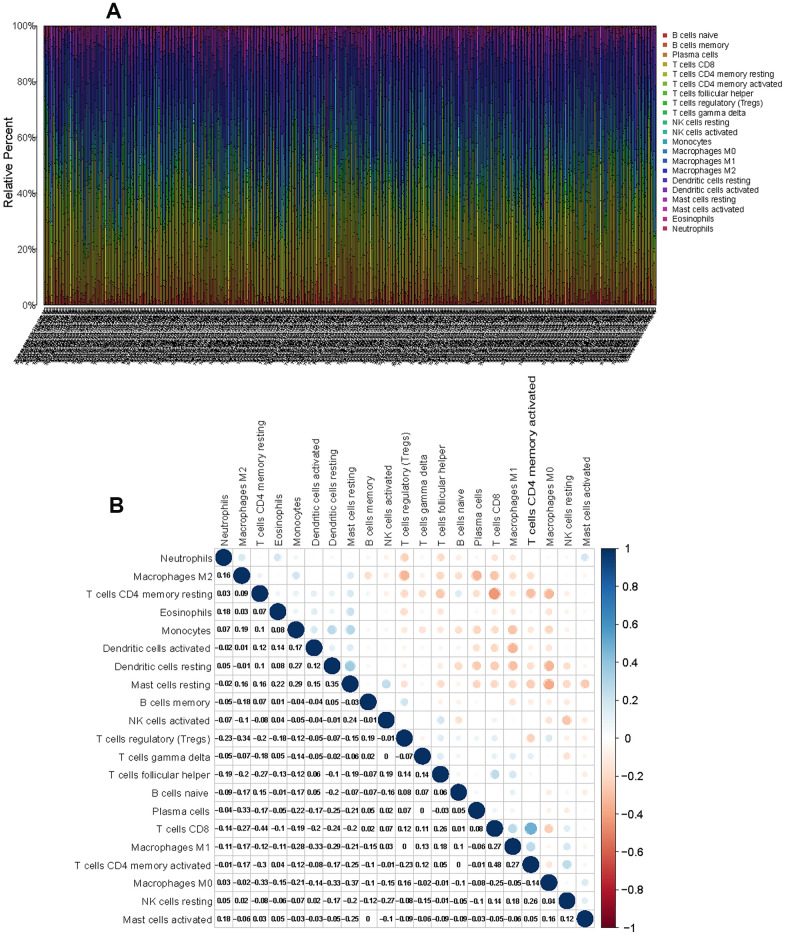
**CIBERSORT for estimating TIIC components in the LUAD microenvironment.** (**A**) Stacked bar chart representing the component of TIICs in LUAD samples. (**B**) Correlation matrix of the different tumor-infiltrating immune cell proportions in LUAD.

To characterize the role played by immune cells in the progression of LUAD, the CIBERSORT algorithm was employed to estimate the differences in immune infiltration of 22 immune cell types in the TME. Figure 6A shows the landscape of the TME immune infiltration model, and every bar plot represents the proportion of 22 immune cells in each sample. Furthermore, the correlation matrix reflected the correlation of different TIICs, ranging from weak to strong, in LUAD (Figure 6B). As shown in the above analysis, CD4 memory resting T cells and CD8 T cells had a strong negative correlation (Cor = -0.44). CD8 T cells exhibited a positive correlation with CD4 memory activated T cells (Cor = 0.48).

### Impact of the expression of the four genes on TIICs in LUAD

**Figure 7:**
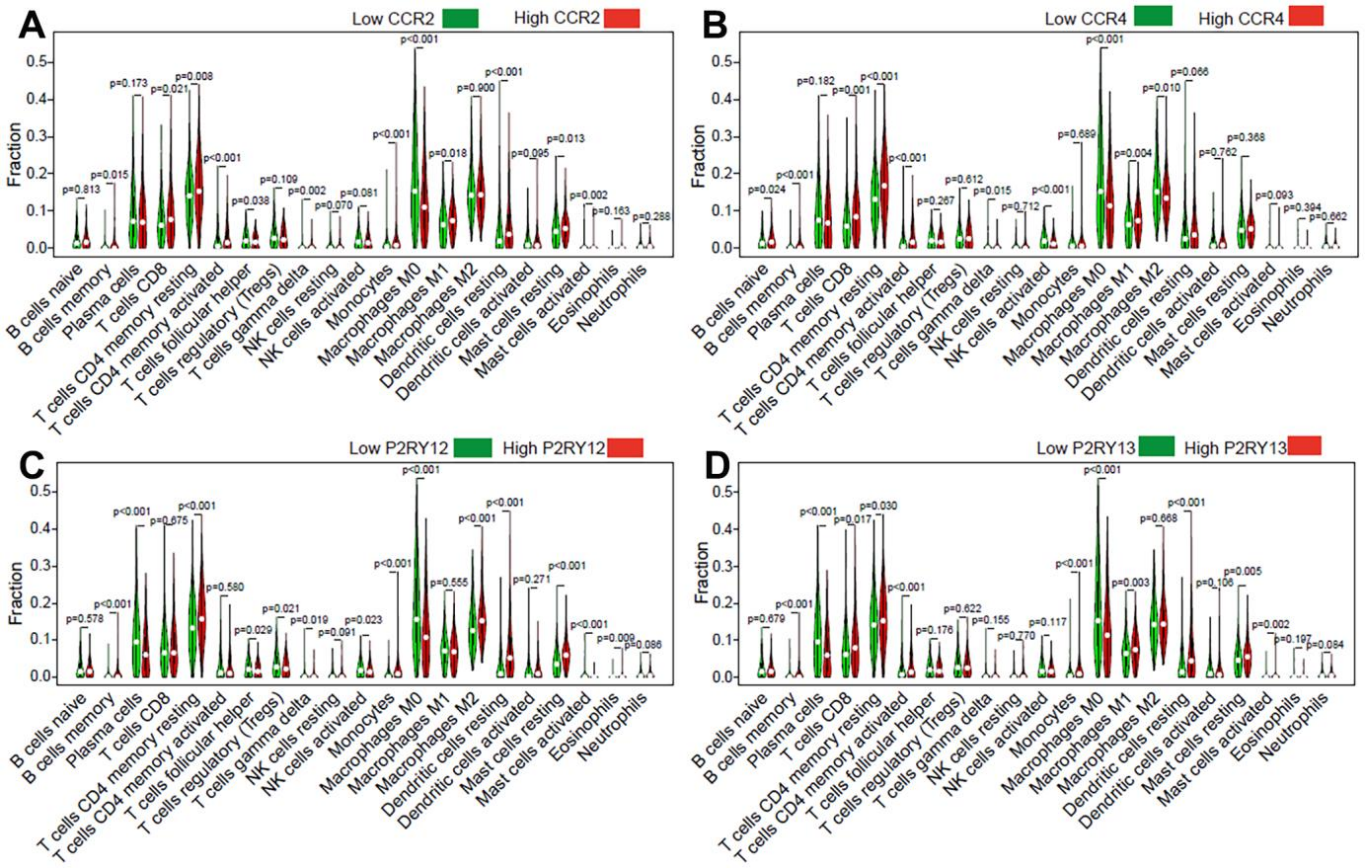
**Effect of the four genes on TIIC levels in patients with LUAD.** Violin plot indicating the ratio differentiation of 21 types of TIICs in high/low CCR2 (**A**), CCR4 (**B**), P2RY12 (**C**), and P2RY13 (**D**) expression relative to the median expression level.

To further clarify the mechanism underlying the functions of the four previously discovered key genes (CCR2, CCR4, P2RY12, and P2RY13) in the tumor microenvironment, we divided LUAD patients into high-expression groups and low-expression groups according to the four gene expression levels. Our results indicated that memory B cells (*p* = 0.015), CD8 T cells (*p* = 0.021), CD4 memory resting T cells (*p* = 0.008), CD4 memory activated T cells (*p* < 0.001), monocytes (*p* < 0.001), M1 macrophages (*p* = 0.018), resting dendritic cells (*p* < 0.001), and resting mast cells (*p* = 0.013) were present in higher proportions in the high CCR2-expression group than in the others (Figure 7A). The relative proportions of follicular helper T cells (*p* = 0.038), gamma delta T cells (*p* = 0.002), M0 macrophages (*p* < 0.001), and activated mast cells (*p* = 0.002) were significantly upregulated in the low CCR2 group (Figure 7A). Naive B cells (*p* = 0.024), memory B cells (*p* < 0.001), CD8 T cells (*p* < 0.001), CD4 memory resting T cells (*p* < 0.001), CD4 memory activated T cells (*p* < 0.001), and M1 macrophages (*p* = 0.004) were present in higher proportions in the high CCR4-expression group than in the low CCR4-expression group, and gamma delta T cells (*p* = 0.015), activated NK cells (*p* < 0.001), M0 macrophages (*p* < 0.001), and M2 macrophages (*p* = 0.01) were significantly upregulated in the low CCR4 group (Figure 7B). Memory B cells (*p* < 0.001), CD4 memory resting T cells (*p* < 0.001), monocytes (*p* < 0.001), M2 macrophages (*p* < 0.001), resting dendritic cells (*p* < 0.001), resting mast cells (*p* < 0.001), and eosinophils (*p* = 0.009) were present in higher proportions in the high P2RY12-expression group than in the others (Figure 7C). The relative proportions of plasma cells (*p* < 0.001), follicular helper T cells (*p* = 0.029), gamma delta T cells (*p* = 0.019), activated NK cells (*p* = 0.023), M0 macrophages (*p* < 0.001), and activated mast cells (*p* < 0.001) were significantly upregulated in the low P2RY12 group (Figure 7C). Memory B cells (*p*<0.001), CD8 T cells (*p*=0.017), CD4 memory resting T cells (*p* = 0.03), CD4 memory activated T cells (*p* < 0.001), monocytes (*p* < 0.001), M1 macrophages (*p* = 0.003), resting dendritic cells (*p* < 0.001), resting mast cells (*p* = 0.005), and activated mast cells (*p* = 0.002) were present in higher proportions in the high P2RY13-expression group than in the low P2RY13-expression groups, and plasma cells (*p* < 0.001) and M0 macrophages (*p* < 0.001) were significantly upregulated in the low P2RY13 group (Figure 7D).

### Correlation analysis of the expression of the four genes with TIICs in LUAD

**Figure 8:**
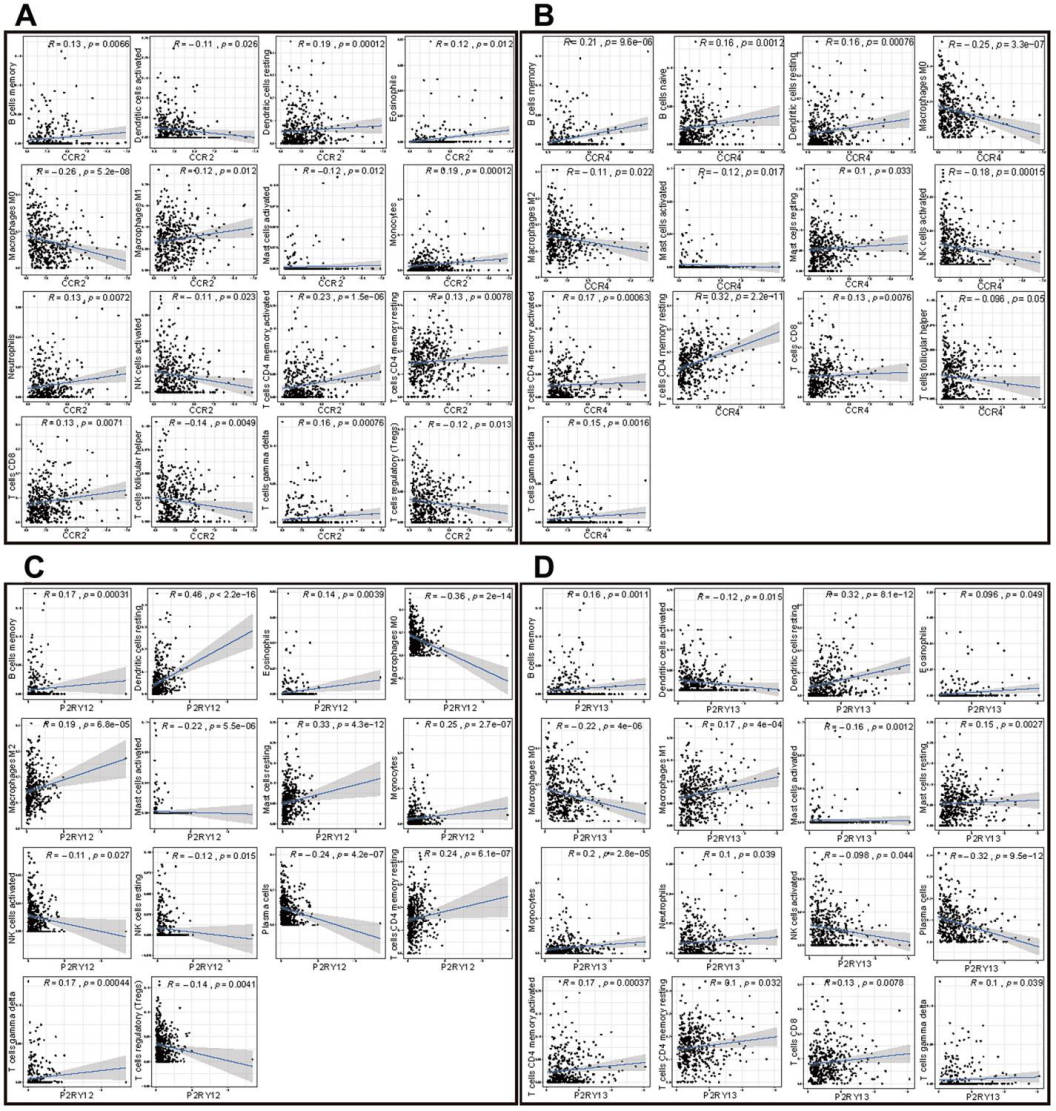
**Correlation of the expression of the four genes with immune cell infiltration levels in patients with LUAD.** (**A**) Scatter plot showing that 16 kinds of TIICs were correlated with the CCR2 expression (*p* < 0.05). (**B**) Scatter plot showing that 13 kinds of TIICs were correlated with CCR4 expression (*p* < 0.05). (**C**) Scatter plot showing 14 kinds of TIICs correlated with P2RY12 expression (*p* < 0.05). (**D**) Scatter plot showing 16 kinds of TIICs correlated with P2RY13 expression (*p* < 0.05).

To further study the relationships among the four genes (CCR2, CCR4, P2RY12, and P2RY13) and immune cell infiltration, we investigated the correlations of the expression of these four genes with TIICs. The TIICs correlated with these four genes are presented in Figure 8. The results indicated that CCR2 was positively correlated with the infiltration of memory B cells, resting dendritic cells, eosinophils, M1 macrophages, monocytes, neutrophils, CD4 memory activated T cells, CD4 memory resting T cells, CD8 T cells, and gamma delta T cells but negatively correlated with the infiltration of activated dendritic cells, M0 macrophages, activated mast cells, activated NK cells, follicular helper T cells, and regulatory T cells (Figure 8A). CCR4 was positively correlated with the infiltration of memory B cells, naive B cells, resting dendritic cells, resting mast cells, CD4 memory activated T cells, CD4 memory resting T cells, and CD8 T cells but was negatively correlated with the infiltration of M0 macrophages, M2 macrophages, activated mast cells, activated NK cells, and follicular helper T cells (Figure 8B). P2RY12 was positively correlated with the infiltration of memory B cells, resting dendritic cells, eosinophils, M2 macrophages, resting mast cells, monocytes, CD4 memory resting T cells, and gamma delta T cells but was negatively correlated with the infiltration of M0 macrophages, activated mast cells, activated NK cells, resting NK cells, plasma cells, and regulatory T cells (Figure 8C). P2RY13 was positively correlated with the infiltration of memory B cells, resting dendritic cells, eosinophils, M1 macrophages, resting mast cells, monocytes, neutrophils, CD4 memory activated T cells, CD4 memory resting T cells, CD8 T cells, and gamma delta T cells but was negatively correlated with the infiltration of activated dendritic cells, M0 macrophages, activated mast cells, activated NK cells, and plasma cells (Figure 8D).

### Screening of immune cells most closely related to the expression of the four genes

**Figure 9:**
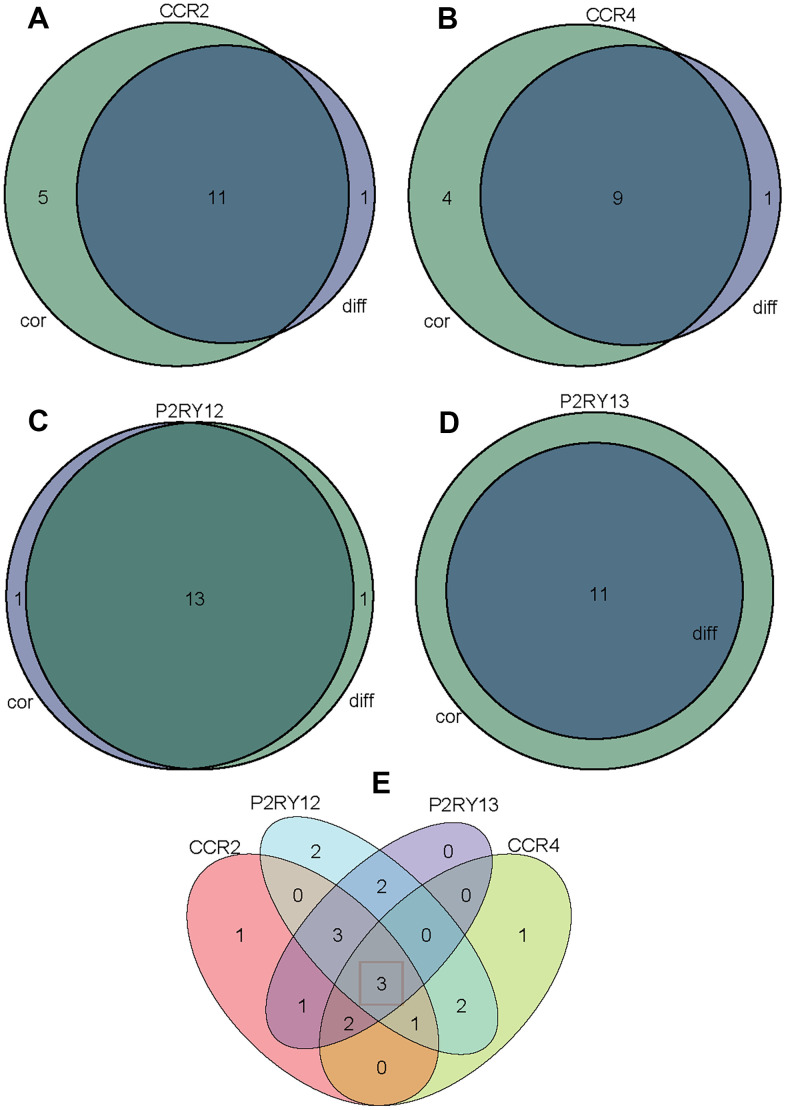
**Venn diagram analysis of aberrantly TIICs based on the difference analysis method and correlation analysis method.** (**A**) Venn diagram indicating 11 kinds of TIICs correlated with CCR2 expression co-determined by difference and correlation analysis displayed in violin and scatter plots, respectively. (**B**) Venn diagram indicating 9 kinds of TIICs correlated with CCR4 expression co-determined by difference and correlation analysis displayed in violin and scatter plots, respectively. (**C**) Venn diagram indicating 13 kinds of TIICs correlated with P2RY12 expression co-determined by difference and correlation analysis displayed in violin and scatter plots, respectively. (**D**) Venn diagram indicating 11 kinds of TIICs correlated with P2RY13 expression co-determined by difference and correlation analysis displayed in violin and scatter plots, respectively. (**E**) Venn diagram indicating 3 kinds of TIICs that were co-related by these four genes.

To screen out immune cells that were most closely related to the expression of the four genes (CCR2, CCR4, P2RY12, and P2RY13), we took the intersection of immune cells differentially infiltrating in high-/low-expression groups and immune cells correlated with the expression of the four genes. Figure 9A indicates that 11 kinds of TIICs correlated with CCR2 expression, which was codetermined by difference and correlation analysis depicted in violin and scatter plots, respectively. Figure 9B indicates that nine kinds of TIICs correlated with CCR4 expression, as co-determined by difference and correlation analysis displayed in violin and scatter plots, respectively. Figure 9C indicates that 13 kinds of TIICs correlated with P2RY12 expression, as co-determined by difference and correlation analysis displayed in violin and scatter plots, respectively. Figure 9D indicates that 11 kinds of TIICs correlated with P2RY13 expression, as co-determined by difference and correlation analysis displayed in violin and scatter plots, respectively. In addition, we took the intersection of all the above TIICs, which indicated that the abundance of memory B cells, CD4 memory resting T cells, and M0 macrophages was jointly regulated by these four genes (Figure 9E).

## DISCUSSION

The percentage of tumor cells determines the purity of the tumor in the TME, which is significantly correlated with the prognosis of cancer patients [23–27]. Rhee et al. determined that tumor purity is an important factor in assessing the correlation between gene expression and clinical pathological features (such as mutation burden, and molecular taxonomy) [26]. It was observed that the purity of glioma is correlated with the main molecular and clinical characteristics of the tumor [27]. Purity-independent subtypes of tumors are closely related to patient prognosis and affect the efficacy of FOLIFIRNOX in the treatment of pancreatic cancer [28]. Immune cells and stromal cells are important components of the tumor microenvironment, affect the purity of tumor cells, and serve anti-tumor functions [29–33].

The ESTIMATE algorithm is employed to calculate the immune score and stromal score based on immune genes and stromal genes in the TME, which can be utilized to reflect the purity of the tumor [34, 35]. In this study, we utilized this algorithm to evaluate the score of stromal and immune-related cells from LUAD patients in the TCGA database. Achieving a better understanding of stromal and immune cells in the TME may establish a foundation for further research characterizing LUAD.

In this research, the immune cells and stromal cells in each sample were scored by ESTIMATE, and the effect of high score or low score on patient prognosis was evaluated. We observed that patients with a high overall score or immune score had a better prognosis and longer survival time than those with a low score. We also analyzed DEGs between patients with high scores and those with low scores. A total of 374 DEGs were employed to perform GO and KEGG pathway enrichment analysis, and it was found that proliferation and activation of immune cells (such as leukocyte proliferation, T cell activation, mononuclear cell proliferation, lymphocyte proliferation, and regulation of lymphocyte activation) were regulated by most of these DEGs. Immune cells regulate tumor behavior and treatment response by interacting with tumor cells with the assistance of cytokines and chemokines [36–39]. Hence, identifying prognostic risk factors related to tumor microenvironmental immunity is highly important for the treatment of tumors. After the Cox regression analysis, 98 DEGs were considered to have significant predictive value for patient prognosis. By intersection of the 98 DEGs and the top 30 genes with the maximum PPI network nodes, four target genes (CCR2, CCR4, P2RY12, and P2RY13) were selected for further study. Except for CCR4, these genes were significantly downregulated in the LUAD immune microenvironment (*p* < 0.05). Interestingly, patients with high expression of these four genes exhibited a better prognosis and longer survival time (*p* < 0.05). To a certain extent, this result also indicated that the immune microenvironment of LUAD is an important factor affecting tumor immunotherapy. Although there are many research reports on screening tumor prognostic markers [40–42], research on the correlation between these markers and TIICs in the TME of LUAD is scarce. To elucidate the mechanism underlying the functions of CCR2, CCR4, P2RY12, and P2RY13 in immune microenvironment of LUAD, we performed GSEA and correlation analysis.

The expression levels of these four genes were closely related to the infiltration of immune cells and the activation or dormancy of immune signaling pathways. We identified the 10 most important signaling pathways enriched in the highly expressed phenotypes of each gene. In addition, we found that the CCR2 expression level was closely related to M0 macrophages, CD4 memory activated T cells, and dendritic cell resting cells. The CCR4 expression level was closely related to memory B cells, CD4 memory resting T cells, and M0 macrophages. The P2RY12 expression level was closely related to dendritic cell resting cells, monocytes, and CD4 memory resting T cells. P2RY13 expression levels were closely related to dendritic cell resting cells, plasma cells, and M0 macrophages. The relative abundances of memory B cells, CD4 memory resting T cells, and M0 macrophages were jointly regulated by these four genes.

It was reported that ablation of CCR2 could inhibit breast cancer bone metastasis by suppressing macrophages [43]. In addition, a CCR4 inhibitor restrained triple-negative breast cancer progression by reducing myeloid-derived immunosuppressor cell recruitment, angiogenesis and metastasis [44]. Most studies have shown that CCR2/CCR4 are highly expressed in tumors and promote tumor progression. Interestingly, and in contrast with the findings of with previous research, our study based on RNA-seq data indicated that LUAD patients with high levels of CCR2 and CCR4 had a better overall survival rate (Figure 4C). These discrepant results may be due to the small sample size. As a family of P2 purinergic receptors (P2RY12), P2RY12 consists of seven transmembrane GPCRs and was reported to be a specific marker for microglial cells. The presence of P2RY12-positive cells was positively correlated with survival rate [45]. P2RY13 is another member of a family of P2 purinergic receptor, which has been reported to be associated with the prognosis of lung cancer [46]. There are few studies on P2RY13 at present. Although P2RY13 has been reported in lung cancer research, this study further investigated the role and possible mechanism of P2RY13 in lung adenocarcinoma from two aspects, namely, of the tumor microenvironment and immune cell infiltration.

To further verify the predictive value of these four genes on the prognosis of lung cancer, the four genes were used to make a cluster. A risk model was calculated based on the expression data of these four genes and Multivariate coefficients. Patients with LUAD were divided into a high-risk group and low-risk group based on the median risk score. The survival curves of patients with high and low risk scores in each subgroup are shown in Supplementary Figure 2, which indicated that patients with high risk score presented a poor survival possibility. All the above records confirmed that the expression of these four genes expression was closely related to the prognosis of LUAD patients and could be utilized as potential markers for the prognosis of lung cancer or targets for the treatment of lung cancer. There are also several limitations to this study. First, this study may have biases resulting from confounding factors due to the lack of wet-lab experiments. Second, the mechanisms by which CCR2, CCR4, P2RY12, and P2RY13 affect immune cell infiltration warrant further study.

In summary, we obtained 318 upregulated genes and 56 downregulated genes by taking the intersection of DEGs between high and low stromal (immune) groups, of which 98 genes might regulate the prognosis of patients with LUAD via Cox regression analysis. The main four target genes (CCR2, CCR4, P2RY12, and P2RY13) were screened out by taking intersection of the top 30 genes with maximum PPI network nodes and 98 candidate prognostic genes. LUAD patients with high expression levels of the four genes had better prognoses and longer survival times. The expression levels of these four genes were closely related to TIICs, which jointly regulated the relative abundances of memory B cells, CD4 memory resting T cells, and M0 macrophages.

## MATERIALS AND METHODS

### Sample and data collection

The transcriptome profiles (n = 551) and clinical data (n = 486) along with adjacent solid tissue normal data for 54 LUAD patients were obtained from publicly available datasets, TCGA, deposited in Genomic Data Commons (GDC) portal (https://portal.gdc.cancer.gov/). All the patients' samples are untreated. Meanwhile, the “estimate score”, “immune score” and “stromal score” in LUAD samples were calculated by the ESTIMATE algorithm using the “estimate” package in R software [47].

### Identification of DEGs and functional enrichment

The R package “limma” was employed to identify DEGs in the immune-score group and stromal-score group in LUAD tissues. To research the biofunctions of DEGs, the R package “clusterProfiler” was used to perform functional annotations, which included three categories of GO (biological processes (BP), molecular functions (MF), and cellular components (CC)) and KEGG enrichment analysis.

### PPI network and cox analysis for screening the four DEGs

To research interactions between the transcription products of these DEGs, we built the PPI network using Cytoscape software and the Search Tool for the Retrieval of Interacting Genes/Proteins (STRING) database. At the same time, Univariate Cox regression analysis was conducted on DEGs to identify 124 candidate prognostic genes with *p*-values less than 0.05. The most important four target genes (CCR2, CCR4, P2RY12, and P2RY13) were identified by taking the intersection of the top 30 genes with the most nodes and 98 candidate prognostic genes.

### Difference analysis and survival curve plotting of the target four genes

Difference analysis of the four genes (CCR2, CCR4, P2RY12, and P2RY13) was performed using the R packages “limma” and “ggpubr”. Survival analysis of these four genes was conducted using the R packages “survival” and “survminer”. The survival curve was plotted using the Kaplan-Meier method, and log rank was used as a significance test.

### GSEA analysis of key prognostic immune-related genes

Gene set enrichment analysis (GSEA) was employed to identify correlation coefficients between biological processed enrichment and the expression of four genes (CCR2, CCR4, P2RY12, and P2RY13). We defined high expression and low expression of these four genes in each cancer type, and then identified the KEGG pathways utilizing GSEA with an adjusted *p*-value < 0.05. The gene sets used in this work were downloaded from the Molecular Signatures Database (MSigDB).

### Characterization of the TME in LUAD

Cell-type identification by estimating relative subsets of RNA transcripts (CIBERSORT) is a method to research cell components of tumor or normal tissues. After normalizing gene expression, the 22 types of infiltrating immune cells (including plasma cells, natural killer cells, 7 T-cell types, macrophages, neutrophils, myeloid subsets, dendritic cells, B cells, among others) were distinguished and deduced the relative proportions by running algorithm CIBERSORT in R in combination with the LM22 signature matrix. Correlation analysis between different TIIC subpopulations was achieved by the "corrplot" package. Twenty-one TIICs between high and low expression of the four genes (CCR2, CCR4, P2RY12, and P2RY13) samples were visualized by the “vioplot” package. CD4 naive cells were excluded because their relative proportion was 0 in all samples. The correlation of the expression of these four genes with the abundance of TIICs was performed by the “limma”, “ggplot2”, “ggpubr”, and “ggExtra” packages.

### Statistical analysis

We used Mann-Whitney U tests or Wilcoxon signed-rank tests to compare gene expression profiles. The Cox, survival, tumor microenvironment, gene difference, and clinical characteristics analyses were carried out using packages implemented in R (v. 3.6.1). The “ggpubr” and “limma” packages were used to validate correlations between the expression of four genes (CCR2, CCR4, P2RY12, and P2RY13) and immune genes. A *p* < 0.05 was considered to be significant. Spearman’s or Pearson’s correlation test was used to evaluate the correlation of two variables. The value of R and *p*-values < 0.05 were the criteria for judging whether there was a correlation.

## Supplementary Material

Supplementary Figures
