# Supplementary material for: Immune profile of the tumor microenvironment and the identification of a four-gene signature for lung adenocarcinoma
Source: Aging (Albany NY). 2020 Dec 9;13(2):2397–417. doi: 10.18632/aging.202269 (PMC7880407; doi:10.18632/aging.202269)
Supplement: Supplementary Figures [file aging-13-202269-s001.pdf]

SUPPLEMENTARY FIGURES

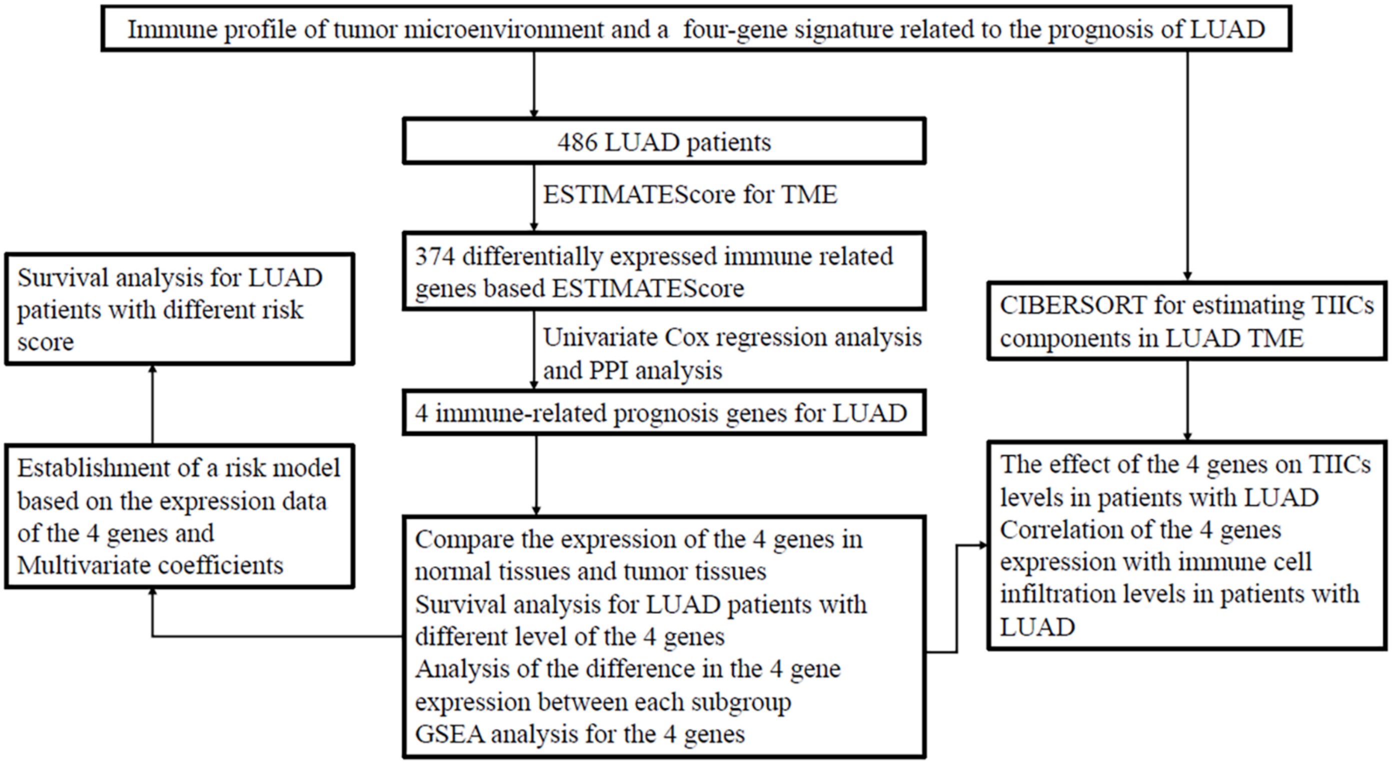

Supplementary Figure 1. **A flowchart showing the screening process.**

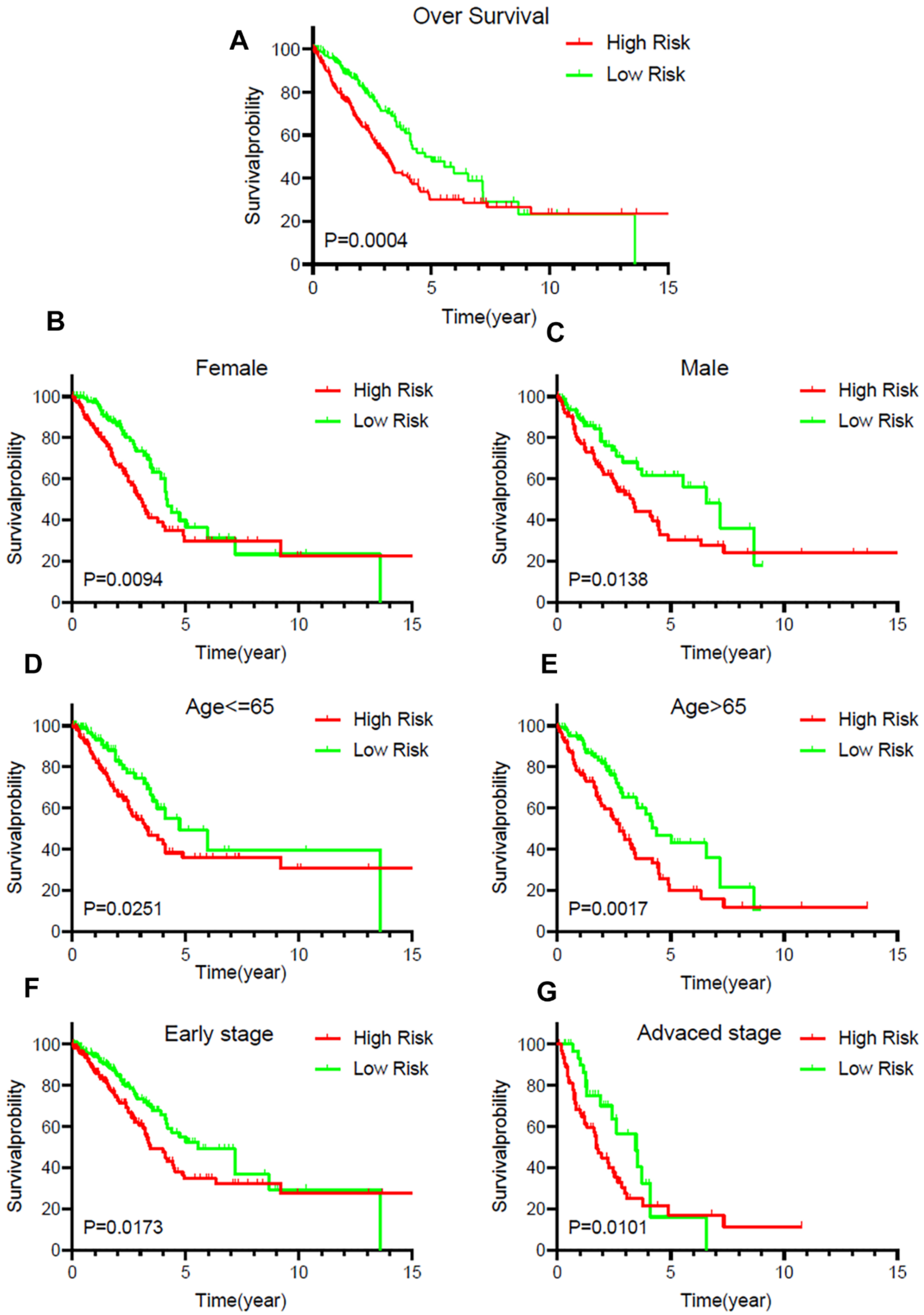

Supplementary Figure 2. Validation the prognostic performance of the four immune related gene signature stratified by overall survival, gender, age and stage. Kaplan-Meier curves for overall survival (**A**), female (**B**), male (**C**), age {less than or equal to} 65 (**D**), age >65 (**E**), early stage (**F**), and advanced stage (**G**) patients based on risk score.
